# Alloreactivity-Based Medical Conditions

**DOI:** 10.1155/2013/567084

**Published:** 2013-06-25

**Authors:** Stanislav Vukmanovic, Margaret G. Petroff, Anne M. Stevens, Daniel Rukavina

**Affiliations:** ^1^Sheikh Zayed Institute for Pediatric Surgical Innovation, Children's National Medical Center, Washington, DC 20010, USA; ^2^Department of Anatomy and Cell Biology, University of Kansas Medical Center, Kansas City, MO 66160, USA; ^3^Department of Pediatrics, University of Washington, Seattle Children's Hospital, Seattle, WA 98109, USA; ^4^Department of Physiology and Immunology, University of Rijeka, 51000 Rijeka, Croatia

Alloreactivity is a response of the immune system to individual antigenic differences within species. These responses in general occur following exposure to alloantigens as a consequence of medical intervention (such as transfusion or tissue transplantation) or during pregnancy. Responses to alloantigens form a basis of a broad spectrum of medical conditions, such as graft rejection, graft versus host disease, reaction to blood (products), or biopharmaceuticals and fetal and neonatal diseases (thrombocytopenia, hemolytic anemia, hemochromatosis, biliary atresia, and glomerulopathy). On the other hand, normal pregnancies are often accompanied by strongly tolerogenic responses to fetal alloantigens. Diverse immunologic components, including T cells, antibodies (B cells), and NK cells, promote alloreactivity, and this complexity makes the pathogenesis and tolerogenesis of these conditions distinct and unique. This special issue contains seven manuscripts touching on various aspects of alloreactivity in medicine.

Blocking CD40-CD40L interaction can induce acceptance of cardiac allografts. However, the blockade is less efficient under inflammatory conditions. In “*Glucocorticoid-induced TNFR-related protein reverses cardiac allograft acceptance induced by CD40-CD40L blockade*,” Krill et al. demonstrate that stimulation of GITR (a cell surface molecule displayed by effector and regulatory T cells) can initiate cardiac graft rejection irrespective of the presence or absence of CD40-CD40L blockade, defining thus at least one possible molecular mechanism of resistance to CD40-CD40L suppression. The balance between the effector and regulatory T cells is critical for development of graft-specific immune responses in general. Franzese et al. review the usefulness of this balance in predicting or establishing the diagnosis of graft rejection in “*Regulatory T cells in the immunodiagnosis and outcome of kidney allograft rejection*.”

Allogeneic hematopoietic stem cell transplantation is used for treatment of autoimmune diseases, multiple organ transplantation, consequences of supralethal irradiation and advanced malignancies and other serious conditions refractory to conventional methods. In “*New allogeneic hematopoietic cell transplantation method: hematopoietic cell transplantation plus thymus transplantation for intractable diseases*,” Hosaka demonstrates that allogeneic hematopoietic stem cell transplantation combined with the same donor thymus transplantation has a superior effect producing more efficient T cell function and reduced graft versus host disease.

Redzovic et al., in “*Mucins help to avoid alloreactivity at the maternal fetal interface*,” review roles of mucin-1 and tumor associated glycoprotein-72 in regulating immune responses. Parallels are drawn between the ability of these molecules to promote tumor growth, invasion, and metastasis and to prevent trophoblast invasion. Removing glycoproteins TAG-72 and Muc 1 from the eutopic implantation site likely contributes to better control of trophoblast invasion by T cells and NK cells. This enables tolerance to paternal antigens of the fetus and normal course and outcome of the pregnancy.

Three manuscripts explore distinct aspects of alloantibody formation. Preformed donor HLA class I-specific antibodies are a risk factor for rejection of kidney, heart, and lung grafts, but the role of these antibodies is less clear in liver transplantation, where rejection rates are lower. Yoshizawa et al., in “*Significance of semiquantitative assessment of preformed donor-specific antibody using luminex single bead assay in living related liver transplantation*,” describe a quantitative assay for donor specific antibodies that has a predictive value for living donor liver transplantation. In “*Nuclear antigens and auto/alloantibody responses: friend or foe in transplant immunology*,” Nakano et al. review current thinking about the role of nuclear antigens and antibodies specific for nuclear antigens in initiation and regulation of immune responses and graft rejection. Finally, transfusion of allogeneic red blood cells may in some recipients induce alloantibodies. Tatari-Calderone et al. in “*The association of CD81 polymorphisms with alloimmunization in sickle cell disease*” suggest that genetic elements in the gene encoding B-cell molecule CD81 may be predictive markers of alloimmunization.

The huge breadth of the field of alloreactivity-based medical conditions allows the special issue only to scratch under the surface of any specific topic. Our hope, however, is that this issue will help raise awareness of the immunological basis of these conditions and will foster further efforts to better understand their pathogenesis. Ultimately, this should help us design better treatments or prevent the development of these medical conditions altogether.

